# Evaluation of immunosuppression protocols for MHC-matched allogeneic iPS cell-based transplantation using a mouse skin transplantation model

**DOI:** 10.1186/s41232-021-00190-7

**Published:** 2022-02-02

**Authors:** Tomoki Kamatani, Ryo Otsuka, Tomoki Murata, Haruka Wada, Takeshi Takahashi, Akihiro Mori, Soichiro Murata, Hideki Taniguchi, Ken-ichiro Seino

**Affiliations:** 1 Division of Immunobiology, Institute for Genetic Medicine, Hokkaido University, Kita-15, Nishi-7, Sapporo, Hokkaido 060-0815 Japan; 2 Central Institute for Experimental Animals (CIEA), Kawasaki, 210-0821 Japan; 3 Department of Regenerative Medicine, Yokohama City University Graduate School of Medicine, 3-9, Fuku-ura, Kanazawa-ku, Yokohama, Kanagawa 236-0004 Japan; 4 Department of Regenerative Medicine, Center for Stem Cell Biology and Regenerative Medicine, The Institute of Medical Science, The University of Tokyo, 4-6-1, Shirokanedai, Minato-ku, Tokyo, 108-8639 Japan

**Keywords:** iPS cell transplantation, Transplantation immunology, MHC-matched, Minor antigen, Immunosuppressive agents, Costimulatory molecule blockade, Humanized mouse

## Abstract

**Background:**

Off-the-shelf major histocompatibility complex (MHC)-matched iPS cells (iPSC) can potentially initiate host immune responses because of the existence of numerous minor antigens. To suppress allo-immune responses, combination of immunosuppressants is usually used, but its efficacy to the allogeneic iPSC-based transplantation has not been precisely evaluated.

**Methods:**

Three transplantation models were used in this study; MHC-matched, minor antigen-mismatched mouse skin or iPSC-graft transplantation, and fully allogeneic human iPSC-derived liver organoid transplantation in immune-humanized mice. The recipients were treated with triple drugs combination (TDC; tacrolimus, methylprednisolone, and mycophenolate mofetil) or co-stimulatory molecule blockade (CB) therapy with some modifications. Graft survival as well as anti-donor T and B cell responses was analyzed.

**Results:**

In the mouse skin transplantation model, immunological rejection caused by the minor antigen-mismatch ranged from mild to severe according to the donor-recipient combination. The TDC treatment could apparently control the mild skin graft rejection when combined with a transient T cell depletion, but unexpected anti-donor T or B cell response was observed. On the other hand, CB therapy, particularly when combined with rapamycin treatment, was capable of attenuating both mild and severe skin graft rejection and allowing them to survive long-term without any unfavorable anti-donor immune responses. The efficacy of the CB therapy was confirmed in both mouse and human iPSC-derived graft transplantation.

**Conclusions:**

The findings suggest that the CB-based treatment seems suitable to well manage the MHC-matched allogeneic iPSC-based transplantation. The TDC-based treatment may be also used to suppress the rejection, but screening of its severity prior to the transplantation seems to be needed.

**Supplementary Information:**

The online version contains supplementary material available at 10.1186/s41232-021-00190-7.

## Introduction

In the field of transplantation medicine, promoting graft acceptance and prolonging graft survival are important issues because of the limited number of donors [1]. Rejection is a major cause of graft loss, and many studies have focused on preventing rejection for decades [2]. Major histocompatibility complex (MHC), or human leukocyte antigen (HLA) in humans, plays an important role in the immune response after allogeneic transplantation as they are expressed on the cell surface and undergo surveillance by recipient immune cells, especially T cells [3]. Therefore, in order to reduce immunological barriers, efforts have been devoted to matching HLA gene haplotypes between donors and recipients of solid organ or bone marrow transplantation [4–6]. Although the main cause of graft loss is HLA mismatch, other antigens, such as minor antigens, can also cause rejection in human [7]. In clinical practice, two large multicenter studies using independent registry data showed decreased long-term survival of kidney transplants performed between HLA haplotype-matched siblings, emphasizing the importance of non-HLA immunity to allogeneic grafts [8, 9]. More recently, genetic mismatch of a kidney-expressed antigen has shown its contribution to a higher risk of rejection in human [10].

The establishment of iPS cells (iPSC) by Takahashi and Yamanaka in 2006 has ushered in a new era for the fields of regenerative medicine, stem cell biology, and drug discovery [11]. In regenerative medicine, cell therapy using iPSC is expected to be a promising solution to donor organ shortage [12]. Practically speaking, however, clinical-grade autotransplantation that meets quality assurance is unlikely to become standard therapy due to its high cost and long preparation time per patient [13, 14]. Therefore, the project has launched to establish well qualified “off-the-shelf” HLA-homozygous iPSC stock, which is considered to be effective at the point of reducing or avoiding immunological rejection [15, 16]. However, rejection caused by minor antigens mismatch remains to be resolved. Our previous study has shown that it was difficult to control rejection of MHC-matched mouse skin grafts even with high-dose tacrolimus (Tac) immunosuppression [17]. In addition, there is a report that MHC-matched iPSC-derived cardiomyocytes transplanted into non-human primates survived for 12 weeks but needed immunosuppressive therapy with methylprednisolone (MP) and Tac [18]. As indicated by these observations, our understanding of the immunological response to MHC-matched iPSC-based allogeneic transplantation and the optimal immunosuppression strategy are still incomplete.

In the present study, we examined the efficacy of traditional combinatory immunosuppressants treatment and of targeted therapy to T cell co-stimulatory molecules on MHC-matched transplantation, measured by the stringent transplantation model of skin grafting. We found that susceptibility to rejection and response to treatment varied among MHC-matched donor-recipient pairs. Conventional immunosuppressants exerted their effect on mild rejection combination while co-stimulatory pathway-directed therapy could well-manage rejection regardless of its severity. We further evaluated these treatment efficacies in mouse and immune humanized mouse transplantation model of iPSC grafts, which presented consistent results to our observation in the skin transplantation model.

## Methods

### Mice

Male BALB/c (haplotype: H2d/d), C57BL/6 J (B6) (H-2b/b), CBA/N (CBA) (H-2 k/k), 129X1/SvJ (H-2b/b), and female C3H/He (H-2 k/k) were purchased from Japan SLC, Inc. (Shizuoka, Japan). Then, 129X1/SvJ males were crossed with C3H/He females to generate male C3129F1 (F1) mice. Nonobese diabetic/Shi-scid, IL-2RγKO Jic (NOG) mice were purchased from In-Vivo Science Inc. (Tokyo, Japan), and immune humanized mice were generated as follows. Male and female NOG mice were irradiated with 160 cGy of X-rays (MBR-1520R-4, Hitachi, Hitachi, Japan), and the umbilical cord blood CD34^+^ cells (5 × 10^4^ cells, StemExpress, Folsom, CA, USA) were transplanted intravenously on the next day. To analyze the hematopoietic chimerism in mice reconstituted with the human immune system, a multicolor flow cytometric analysis was performed using BD LSRFortessa (BD Biosciences, Franklin Lakes, New Jersey, USA). The peripheral blood was periodically collected from the retro-orbital venous plexus using capillary pipettes with sodium heparinization (Paul Marienfeld GmbH & Co.KG, Lauda-Königshofen, Germany) under anesthesia with isoflurane every 4 weeks. Red blood cells were lysed using an ammonium-chloride-potassium solution (150 mM NH4Cl, 10 mM KHCO3, and 1 mM EDTA-Na2), and the mononuclear cells were stained with antibodies for flow cytometry. Human CD45 chimerism was calculated using human CD45^+^ cells relative to the total CD45^+^ cells, which included the human and mouse CD45^+^ cell populations.

All animal procedures were approved by the Hokkaido University Animal Care Committee (approval number: 18-0004) and the Central Institute for Experiment and Yokohama City University’s animal experiment committee (approval number: F-A-17-025, F-A-20-021). All animal experiments were performed in accordance with the ARRIVE (Animal Research: Reporting of In Vivo Experiments) guidelines.

### Cell culture

Mouse iPSC were maintained on laminin-coated culture dish with maintenance medium consisted of advanced-DMEM/F-12 (1:1 mixed, Thermo, SIGMA) supplemented with 0.5 × Neuro Brew-21 (Miltenyi), 0.5 × N2 supplement (Wako), and 10 U/ml penicillin, 100 μg/ml streptomycin, 0.1 mM 2-mercaptoethanol (Nacalai), 0.03% l-glutamine (Gibco), 3 μM CHIR99021 (Adooq), 1 μM PD0325901 (Tocris), and recombinant human leukemia inhibitory factor (produced in our laboratory). Fibroblasts were maintained on gelatin-coated culture dish with DMEM (Wako) supplemented with 10% fetal bovine serum (Sigma), 0.1 mM non-essential amino acids, 1 mM sodium pyruvate, and 10 U/ml penicillin, 100 μg/ml streptomycin. Cells were maintained in a 5% CO2/air environment at 37 °C.

### Luc-iPSC generation

CBA and B6-derived iPSC were generated from fibroblasts isolated from fetal CBA and B6 mice and validated by previously described methods [19]. Luciferase (Luc) lentiviral particles were generated by transfecting Lenti-X 293 T cells with psPAX2 (Addgene, #12260), pMD2.5 (Addgene, #12259), and pHIV-Luc-ZsGreen (Addgene, #39196) using polyethylenimine max. Supernatants containing lentiviral particles were collected and used to infect mouse iPSC, which were then continuously selected by ZsGreen expression.

### Human iPSC-derived liver organoids generation

The generation of human iPSC-derived liver organoids was carried out as described previously [20]. We collected hepatic endoderm, endothelial, and mesenchymal cells and seeded them on Elplasia six-well plates (Corning, Corning, NY, USA). The seeded cell number for hepatic endoderm was 2.5 × 10^6^ cells, for endothelial, 1 × 10^6^ cells, and, for mesenchymal cells, 1 × 10^6^ cells per well. The culture medium used was the same as reported previously [21]. Y-27632 (FUJIFILM Wako Pure Chemical Corporation, Osaka, Japan) was added on day one. On day two, small liver organoids were collected and reseeded on a cell culture insert (Corning, Corning, NY, USA). The medium was changed every other day.

### Transplantation

For mouse skin transplants, recipient mice were anesthetized by intraperitoneal injection of a three-drug mixture of medetomidine (Domitor Nippon Zenyaku Kogyo Co., Ltd.), midazolam (midazolam injection, TEVA, Takeda Pharmaceutical Co., Ltd.), and butorphanol (Vetorphale, Meiji Seika), and the dorsal side of the auricle skins from donor mice were transplanted into dorsal thoracic of the recipients. Recipient mice were wrapped with bandages for 7 days to protect skin grafts and warmed up until they moved freely as previously described [22]. To assess rejection, the graft diameter was measured. The day of graft rejection was determined as the day on which the graft diameter reached less than 30% of the initial diameter or the recipients died. Graft survival was calculated using the following formula: (total number of transplanted grafts − number of rejected grafts)/total number of transplanted grafts × 100. All the mice were euthanized by cervical dislocation at the end of experiments.

For mouse iPSC-derived Luc-iPSC-graft transplants, recipient mice were anesthetized by intraperitoneal injection of a three-drug mixture of medetomidine, midazolam, and butorphanol, and 2.5 × 10^6^ CBA or B6-derived Luc iPSC were injected into the gastrocnemius muscle of recipient mice. Transplanted cell survival was longitudinally monitored via in vivo bioluminescent imaging (BLI) by using IVIS Spectrum Imaging Systems (Spectrum-FL-TKHD; Caliper Life Sciences Ltd.) (POD0, 2, 4, 6, 8, 11, 13, 17, 21, 25). d-Luciferin (Cayman) was administered intraperitoneally at a dose of 120 mg/kg of body weight. After 20 min from the administration of d-Luciferin, recipient mice were placed in a light-tight chamber, and photons emitted from Luc-expressing cells were collected with integration times of 20 s. BLI signal was quantified in maximum photons per second per centimeter square per steradian (p/s/cm^2^/sr) and presented as log10 [photons per second]. All the mice were euthanized by cervical dislocation at the end of experiments.

For liver organoids transplants, the mice were anesthetized by isoflurane and the opened peritoneum. The mesothelium of the left liver lobe was peeled off, and iPSC-derived liver organoids were transplanted onto the peeled-off portion. The transplanted cell number was 2 × 10^6^ cells in hepatic endoderm equivalents. After the transplantation, the transplant was covered with the middle lobe of the liver and the closed peritoneum. All the mice were euthanized by cervical dislocation at the end of experiments [23].

### Immunosuppressants

For the conventional triple drugs combination (TDC) treatment, tacrolimus (Astellas, Japan), methylprednisolone (Cayman, #15013), and mycophenolate mofetil (CHUGAI PHARMACEUTICAL CO., Ltd., Japan) were administered once daily by intraperitoneal injections, 0.5 mg/kg/day for tacrolimus, 5 mg/kg/day for methylprednisolone, and 100 mg/kg/day for mycophenolate mofetil. For T cell depletion, anti-CD4 (GK1.5) and CD8 (53-6.72) monoclonal antibodies (mAb) (purified in our laboratory) were administered by intraperitoneal injections on days -6, and -1 post-transplantation days. For co-stimulatory molecule blockade (CB) therapy, anti-CD40 ligand (CD40L) (MR-1 for mouse and #BE0292 for human, BioXcell) and CTLA4Ig (Orencia®, Bristol Myers Squibb for mouse and #BE0099, BioXcell for human) were administered at a dose of 500 μg for anti-CD40L, 400 μg for CTLA4Ig on days 0, 2, 4, and 6 after transplantation. Rapamycin (LC Laboratories, R-5000) were administered 2.0 mg/kg by intraperitoneal injections once every 3 days starting the day before transplantation. We have determined the dosage of immunosuppressants referring to previous reports [24–27]. Especially for TDC, we made the dosage of them more than those used in human (per kilogram). We have measured bodyweight of the treated mice and found that there was no abnormal weight-loss in each treatment group (Additional file 1: Supplementary Fig. 4E-H).

### HE and immunohistochemistry

The skin grafts were fixed in 4% paraformaldehyde and embedded in paraffin. The blocks were sliced into 5-μm sections and stained with anti-mouse CD3ε (A0452, Dako, Glostrup, Denmark) as the primary antibody and then with anti-rabbit IgG-horse radish peroxidase (K4003, Dako) as a secondary antibody. Antibody binding was detected with a chromogenic substrate for horseradish peroxidase. The samples were counter stained with hematoxylin and eosin (HE) or Kernechtrot (Merck Millipore, Billerica, MA, USA).

### Flow cytometry and antibodies

Flow cytometry was performed using FACSCelesta (BD Biosciences), SH800S (SONY), or FACSAria II (BD Biosciences), and the data were analyzed by the FlowJo software V10.7.1 (Tree Star, Ashland, OR, USA). The antibodies used in FACS were listed in Additional file 2: Supplementary Table 1. For analysis, live cells were gated based on forward and side scatter as well as a lack of propidium iodide (PI) or 4′,6-diamidino-2-phenylindole (DAPI) uptake.

### Mixed lymphocyte reaction

To assess the recipients’ T cell response, we performed a mixed lymphocyte reaction as follows. Recipient mice fresh whole splenocytes were used as responder cells. As stimulator cells, 30 Gy-irradiated fresh whole splenocytes from BALB/c, B6, CBA, or F1 were used. Carboxyfluorescein diacetate succinimidyl ester (CFSE) (Dojindo Laboratories, Kumamoto, Japan)-labeled responder cells (2 × 10^5^) were co-cultured with stimulator cells (8 × 10^5^) in 96-well round-bottomed culture plates in RPMI-1640 high-glucose supplemented with 10% fetal bovine serum, 0.1 mM non-essential amino acids, 1 mM sodium pyruvate, 50 μM 2-mercaptoethanol, 100 U/mL penicillin, and 100 μg/mL of streptomycin (all from Life Technologies, Carlsbad, CA, USA). On day 4 of culture, T cell proliferation was assessed by measuring the reduction in CFSE fluorescence by flow cytometry.

### Measurement of alloantibody production

In vivo antibody production was analyzed as previously described [22]. Briefly, 100 days after skin graft transplantation, the sera were harvested from F1 recipient mice by blood collection. To detect antibodies against the donor antigen in the sera, thymocytes from BALB/c, B6, CBA, or F1 mice Fc receptor were blocked and then incubated with the sera. After washout of unbound antibodies, the cells were further incubated with Alexa fluor-488-conjugated goat anti-mouse IgG antibody and PE anti-mouse CD19 and then were analyzed with an FACSCelesta and FlowJo software.

### Human albumin quantification

Quantification of human albumin was carried out as described previously [21]. All blood samples of the mice were collected before transplantation and then were collected every week after transplantation. Blood samples were centrifuged by 4000 rpm, 20 min at 4 °C, and the plasma was collected. The collected plasma was diluted, and the human albumin concentrations were assessed by enzyme-linked immunosorbent assay (Bethyl Laboratories, Montgomery, TX, USA), according to the manufacturer’s instructions.

### Statistical analyses

Statistical data were analyzed with the JMP software (JMP Pro Version 15.2.0, SAS Institute, Inc., Cary, NC, USA; JMP license was distributed by Hokkaido University Graduate School of Medicine.). Kaplan-Meier survival curves were analyzed using the log-rank test or Tukey’s honestly significant difference (HSD) test. *P* values less than 0.05 were considered to indicate statistically significant results.

## Results

### Application of standard immunosuppressive therapy in human solid organ transplantation to MHC-matched mouse skin transplantation

**Fig. 1:**
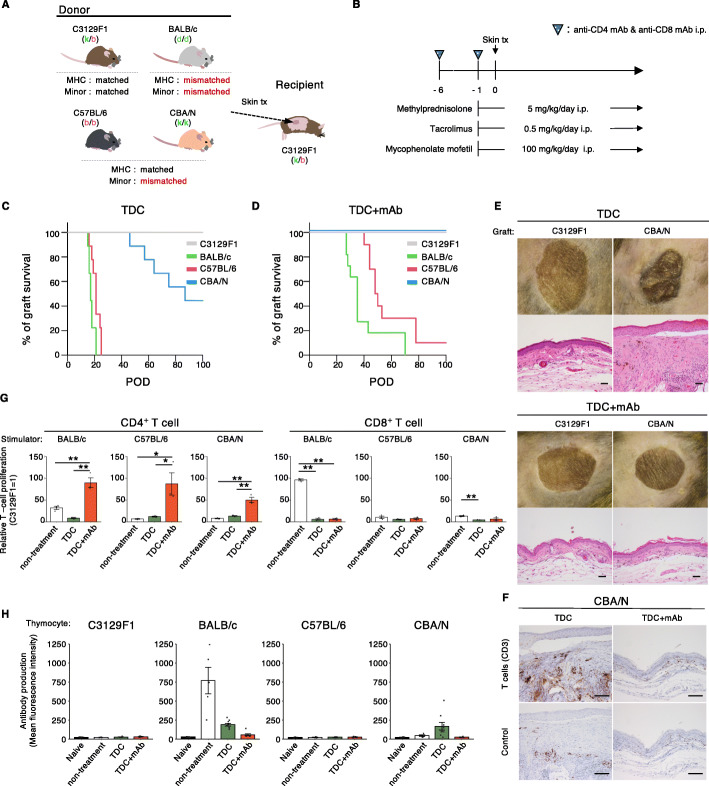
Standard immunosuppressive therapy in solid organ transplantation has limited efficacy in mouse skin transplantation, even in MHC-matched situations. (**A**) Schematic overview of auto or allogeneic skin transplantation. (**B**) TDC-based immunoregulation protocol. Immunosuppression was performed by using a protocol of administrating a combination of methylprednisolone, tacrolimus, and mycophenolate mofetil daily (TDC) with or without anti-CD4 mAb and anti-CD8mAb on day 6 and 1 (TDC mAb). (**C**) Skin graft survival under immunosuppression with TDC (*n* = 9, C3129F1; *n* = 9, BALB/c; *n* = 9, C57BL/6; *n* = 9, CBA/N). (**D**) Skin graft survival under immunosuppression with TDC + mAb (*n* = 10, C3129F1; *n* = 11, BALB/c; *n* = 10, C57BL/6; *n* = 9, CBA/N). (**E**) Macroscopic observation (upper panels) and hematoxylin and eosin stained section (lower panels) of autologous (C3129F1) and CBA/N skin grafts on day 100. Scale bars: 100 μm. (**F**) Immunohistochemical staining for CD3 of CBA/N skin grafts harvested from TDC or TDC + mAb treated groups on 100 days post-transplantation. Upper and lower panels represent CD3 specific staining and isotype control stained section, respectively. Scale bars: 100 μm. (**G**) Recipient T cell response in MHC-matched but minor antigen-mismatched skin transplantation. The T cell proliferation rates in each treatment group were normalized to that of C3129F1 mice stimulated with autologous irradiated splenocytes. Error bars indicate standard error of technical triplicates. Similar results were obtained in two independent experiments. *p < 0.05, **p < 0.01 (Tukey’s HSD test). (**H**) De novo anti-donor antibody production in the recipients. Error bars indicate standard error of biological replicates (*n* = 8, naive; *n* = 4-5, non-treatment; *n* = 9, TDC; *n* = 7, TDC + mAb). tx, transplantation; mAb, monoclonal antibody; TDC, three drug combination; POD, post-operative day

We first attempted to control rejection in an MHC-matched mouse skin graft model, which imitates an iPSC-based tissue transplantation, by conventional immunosuppressive therapy commonly used in clinical solid organ transplantation. MHC homo-to-hetero skin transplantation was performed using a method based on our previous report [17] where F1 (H-2b/k) mice were grafted with skin from B6 (H-2b/b), CBA (H-2 k/k), or BALB/c (H-2d/d) mice (Fig. 1A). Without any treatment, the grafts from every donor were completely rejected within 27 days [17]. Recipients were treated with a calcineurin inhibitor (tacrolimus, Tac), inosine monophosphate dehydrogenase inhibitor (mycophenolate mofetil, MMF), and corticosteroid (methylprednisolone, MP), that have long been combinatory used to control rejection in human MHC-mismatched transplantation (Fig. 1B) [28]. In the triple drugs combination (TDC) group, recipient F1 mice rejected MHC-mismatched BALB/c skin grafts within 21 days with a median survival time (MST) of 17 days (Fig. 1C). Similarly, MHC-matched but minor antigen-mismatched B6 skin grafts were rejected within 25 days (MST = 21 days) (Fig. 1C). On the other hand, more than 40% (4/9) of skin grafting from MHC-matched but minor antigen-mismatched CBA to F1 remained accepted on 100 days post-transplantation (Fig. 1C). Although we have previously demonstrated that high dose treatment (2 mg/kg/day) of Tac monotherapy was not effective enough to control rejection against B6 and CBA skin grafts in this model [17], combinatory administration resulted in prolonged CBA skin engraftment. However, macroscopic observation of survived CBA skins on day 100 exhibited epidermal atrophy compared to autologous grafts, and HE staining analysis of paraffin-embedded sections revealed skin graft hyperplasia and immune cell infiltration. We further analyzed graft infiltrated cells by immunostaining, revealing CD3^+^ cell infiltration in the TDC-treated CBA skin grafts (Fig. 1E and F). In line with our previous observation, these results suggest that the severity of graft rejection in MHC homo-to-hetero transplantation under the TDC treatment varies from mild (e.g., CBA) to severe (e.g., B6) depending on the combination of donor and recipient.

### Transient T cell depletion significantly improved the efficacy of TDC treatment, particularly for the mild rejection

Usually, tissue or cellular grafts are suffered from severe rejection than solid organ grafts in mouse allogeneic transplantation [29]. In pancreatic islet transplantation in human, addition of anti-thymocyte globulin to conventional immunosuppression significantly improved the outcome [30]. Therefore, we next investigated mouse skin graft survival with a transient T cell depletion by anti-CD4 and CD8 monoclonal antibodies (mAb) as preconditioning prior to TDC therapy (TDC + mAb) (Fig. 1B). As a result, rejection of CBA skin graft was not observed, and all grafts survived more than 100 days (Fig. 1D). In addition, the MST of BALB/c and B6 skin showed improvement to 35 and 49 days, respectively, and 10% (1/10) of B6 grafts reached 100-day survival (Fig. 1D). Visual observation of survived CBA skin grafts on day 100 showed no epidermal atrophy, and HE staining showed the similar observation image as autologous grafts. In addition, immunostaining showed no significant infiltration of CD3^+^ cells into the TDC + mAb-treated CBA skin grafts (Fig. 1E and F).

### Evaluation of anti-donor T and B cell responses in TDC-based treatment

We performed mixed lymphocyte reaction (MLR) using splenocytes from the recipient mice of each treatment group collected later than 100 days post-transplantation. Recipient splenocytes were labeled with CFSE and co-cultured with irradiated donor splenocytes. As a result, the proliferation rate of CD8^+^ T cells against BALB/c was prominent which was dramatically decreased in the treated groups, while the proliferation rate against B6 and CBA in non-treatment group was relatively low, and suppression effect by TDC-based treatment was hardly observed (Fig. 1G). On the other hand, significant CD4^+^ T cell proliferation was observed against every donor in the TDC + mAb group, while no change was observed in the TDC alone group compared to the non-treatment group (Fig. 1G). The drastic proliferation of CD4^+^ T cells in the TDC + mAb group would reflect the emergence of in vivo formation and activation of allo-specific memory T cells, which were suggested to occur following transient T cell depletion in both mouse and human [31–33]. Although memory T cells can conduct graft rejection, continuous tacrolimus administration may have effectively suppressed their activation as indicated previously in clinical cases [34], and consequently mild-combination (CBA) grafts survived long-term.

We also investigated the production of donor-reactive antibodies, which was reported to be one of the causes of chronic rejection [35–37]. As a result, sera collected from the non-treatment group did not contain anti-B6 or anti-CBA antibodies consistent with our previous observation [17] (Fig. 1H). Interestingly, analysis of sera from TDC-treated recipients indicated the presence of CBA reactive antibody while it was abolished by transient T cell depletion (Fig. 1H). MHC-mismatched BALB/c provoked substantial antibody production in the non-treatment group, but TDC treatment decreased that level, and transient T cell depletion minimized anti-BALB/c antibody production (Fig. 1H). Considering the detection of antibodies against CBA but not B6 in TDC-treated group, our findings suggest that prolonged graft survival might elicit antibody production even in MHC-matched transplantation, and the T cell presence at the peri-transplantation period may have a significant role in anti-minor antigen antibody production.

The above results suggest that transient T cell depletion enables TDC therapy to regulate MHC homo-to-hetero transplantation in the mild-combination setting in terms of no significant infiltration of immune cells into the grafts and inhibition of antibody production. However, robust CD4^+^ T cell proliferation in MLR would be reminiscent of memory T cell formation triggered by the transient T cell depletion, and it could not significantly suppress the CD8^+^ T cell proliferation compared to the non-treatment group. In addition, in severe-combination settings like B6 to F1, the impact of T cell depletion was marginal. Therefore, TDC-based immune suppression seemingly achieved long-term graft survival but may remain to be improved, particularly evaluating their combinatory usage with other therapeutic reagents.

### Blockade of leukocyte co-stimulatory molecules permits long-term engraftment of mouse skin grafts from MHC-matched allogeneic donors

**Fig. 2:**
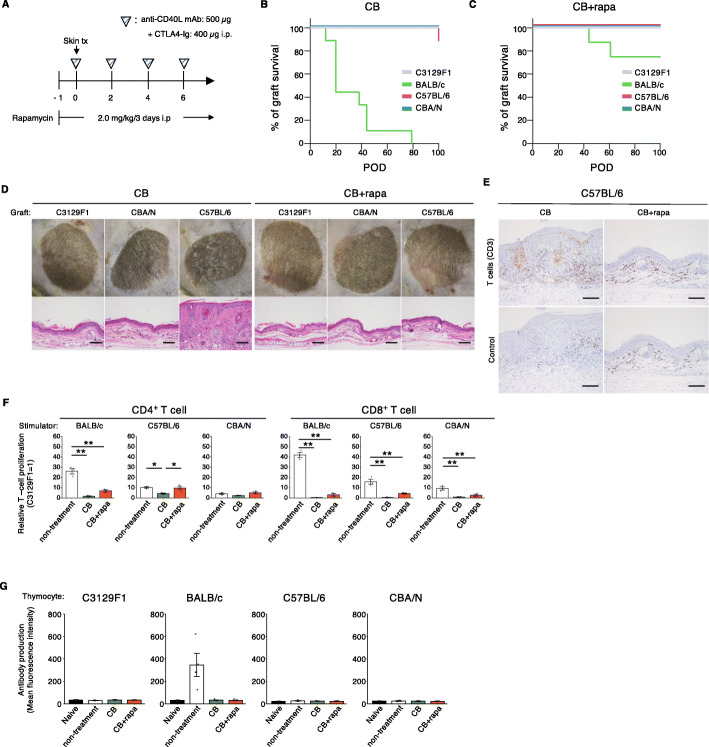
Blockade of leukocyte costimulatory molecules permits long-term engraftment of mouse skin grafts from MHC-matched allogeneic donors. (**A**) CB-based immunoregulation protocol. Immunosuppression was performed by using a protocol of administrating a combination of anti-CD40L mAb and CTLA4-Ig daily (CB) with or without rapamycin on every 3 days from POD 1 (CB + rapa). (**B**) Skin graft survival under immunosuppression with CB (*n* = 9, C3129F1; *n* = 9, BALB/c; *n* = 9, C57BL/6; *n* = 9, CBA/N). (**C**) Skin graft survival under immunosuppression with CB + rapa (*n* = 9, C3129F1; *n* = 8, BALB/c; *n* = 9, C57BL/6; *n* = 9, CBA/N). (**D**) Macroscopic observation (upper panels) and hematoxylin and eosin stained section (lower panels) of autologous (C3129F1), CBA/N, and C57BL/6 skin grafts on day 100. Scale bars: 100 μm. (**E**) Immunohistochemical staining for CD3 of C57BL/6 skin grafts harvested from CB or CB + rapa treated groups on 100 days post-transplantation. Upper and lower panels represent CD3 specific staining and isotype control stained section, respectively. Scale bars: 100 μm. (**F**) Recipient T cell response in MHC-matched but minor antigen-mismatched skin transplantation. The T cell proliferation rates in each treatment group were normalized to that of C3129F1 mice stimulated with autologous irradiated splenocytes. Error bars indicate standard error of technical triplicates. Similar results were obtained in two independent experiments. *p < 0.05, **p < 0.01 (Tukey’s HSD test). (**G**) De novo anti-donor antibody production in the recipients. Error bars indicate standard error of biological replicates (*n* = 8, naive; *n* = 4, non-treatment; *n* = 9, CB; *n* = 9, CB + rapa). tx, transplantation; mAb, monoclonal antibody; CB, co-stimulatory molecule blocking; rapa, rapamycin; POD, post-operative day

Co-stimulatory molecules such as CD28 or CD40L on T cell surface conduct activation signals through binding to their counterpart on antigen-presenting cell (APC), and their inhibition has been experimentally shown to be effective in controlling allograft rejection. We next attempted to control rejection by combinatory administering co-stimulatory molecule blocking (CB) reagents (Fig. 2A). In the treatment group with CB, recipient F1 mice rejected MHC-mismatched BALB/c skin grafts within 79 days (MST = 20) (Fig. 2B). On the other hand, both CBA and B6 grafts were mostly viable at 100 days post-transplantation (Fig. 2B). Additionally, we attempted to control the rejection of skin grafts by supplementing rapamycin (mTOR inhibitor) maintenance therapy in addition to CB (CB + rapa) (Fig. 2A). We have previously reported that rapamycin monotherapy could not induce long-term graft acceptance in this skin transplantation model [17]. The results showed that 100% of the grafts were viable at 100 days post-transplantation in both CBA and B6 to F1 transplantation (Fig. 2C). In this setting, 75% of BALB/c grafts were viable at 100 days post-transplantation (Fig. 2C).

We have directly compared the effect of TDC- and CB-based treatments in the mouse skin transplantation model. The graft survival data with respect to each donor strain were shown in Additional file 1: Supplemental Fig. 4A-D. Both TDC + mAb and CB + rapa treatments induced long-term graft acceptance (> 100 days) in CBA donor, while only CB + rapa but not TDC + mAb induced it in B6 donor (MST for TDC + mAb in B6 was 49 days). Similarly, in the group pf BALB/c donor which was MHC-mismatched, all the grafts were completely rejected within 70 days with TDC + mAb treatment, while 75% (6/8) grafts were accepted more than 100 days with CB + rapa treatment. Therefore, we concluded that CB + rapa was more effective to induce long-term graft acceptance than TDC + mAb in the model of mouse skin transplantation.

Macroscopic observation of the CB-treated skin grafts on day 100 indicated that the CBA skin grafts did not show epidermal atrophy as in the autografts, while the B6 skin grafts showed partial tissue damage (Fig. 2D). In addition, HE staining revealed CB-treated B6 skin grafts underwent hyperplasia accompanying CD3^+^ cell infiltration, whereas CB + rapa treatment inhibited T cell infiltration and showed normal skin histology (Fig. 2D and E). The MLR results showed that the proliferation rates of CD8^+^ T cells in both treatment groups were significantly decreased compared with non-treatment group (Fig. 2F). CD4^+^ T cell proliferation levels against B6 and CBA were mainly comparable to those of the non-treatment group regardless of long-term engraftment. Interestingly, CB-based treatment significantly suppressed CD4^+^ and CD8^+^ T cell proliferation even against MHC-mismatched BALB/c splenocytes, which was not observed in TDC-based treatment, particularly for CD4^+^ T cells. We then tested for the presence of donor-specific antibody production in the serum of recipient mice treated with CB or CB + rapa, but no donor-specific antibodies were detected except for anti-BALB/c in the non-treatment group (Fig. 2G). Therefore, immunomodulation targeting the co-stimulation pathway in MHC-matched tissue allografting, particularly when reinforced with rapamycin maintenance treatment, can control graft rejection without any immunological signs such as robust T cell proliferation in MLR and antibody production.

### Co-stimulatory blockade effectively prevents rejection to iPSC-grafts

**Fig. 3:**
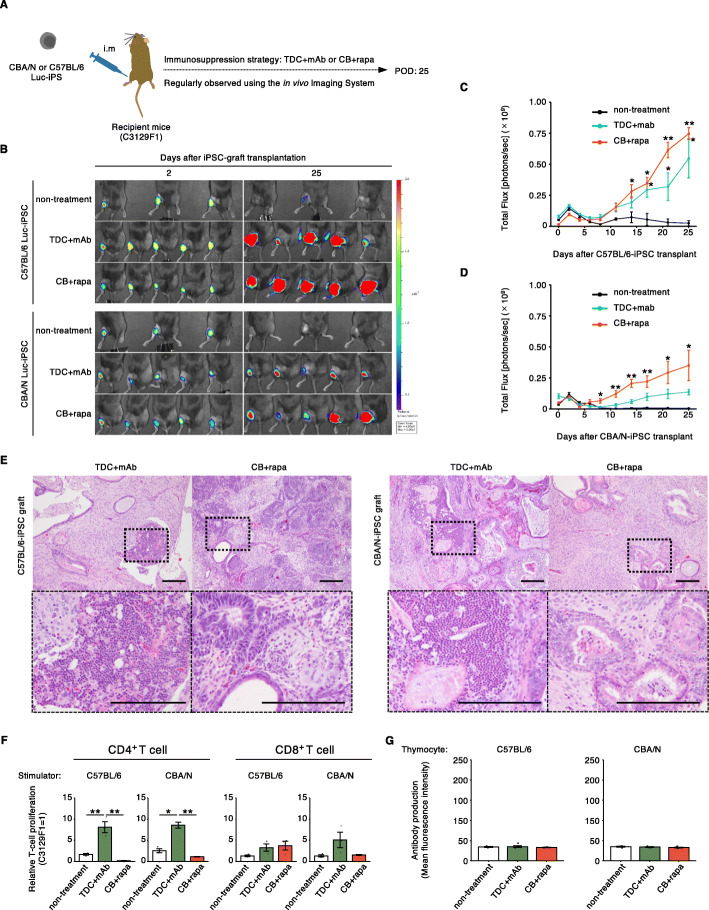
Co-stimulatory blockade effectively prevents rejection to iPSC-grafts. (**A**) Schematic overview of CBA/N or C57BL/6 Luc-iPSC transplantation. (**B**) Bioluminescence images of C57BL/6 (upper panels) or CBA/N (lower panels) Luc-iPSC transplanted mice that received non-treatment, TDC + mAb, or CB + rapa. *n* = 3-5 per group. (**C**, **D**) Quantitative bioluminescence intensity of C57BL/6 (**C**) or CBA/N (**D**) Luc-iPSC-transplanted mice that received non-treatment, TDC + mAb, or CB + rapa. *n* = 3-5 per group. *p < 0.05, **p < 0.01 (Tukey’s HSD test). (**E**) Hematoxylin and eosin stained sections of C57BL/6 (left panel) or CBA/N (right panel) Luc-iPSC-derived teratomas under immunosuppression with TDC + mAb or CB + rapa on day 35. Lower panels show enlargement of insets in the upper panels. Scale bars: 100 μm. (**F**) Recipient T cell response in MHC-matched but minor antigen-mismatched iPSC transplantation. The T cell proliferation rates in each treatment group were normalized to that of C3129F1 mice stimulated with autologous irradiated splenocytes. Error bars indicate standard error of technical triplicates. Similar results were obtained in two independent experiments. *p < 0.05, **p < 0.01 (Tukey’s HSD test). (**G**) De novo anti-donor antibody production in the recipients. Error bars indicate standard error of biological replicates (*n* = 3, non-treatment; *n* = 5, TDC + mAb; *n* = 5, CB + rapa). Luc, luciferase; TDC, three drug combination; mAb, monoclonal antibody; CB, co-stimulatory molecule blocking; rapa, rapamycin; POD, post-operative day

We next attempted to control the immune response in the transplantation of iPSC in MHC-matched/minor antigen-mismatched situation. The iPSC used for the experiments were generated from embryonic fibroblasts of B6 or CBA mice and then were transduced with firefly luciferase (Luc) gene to monitor iPSC survival by detecting bioluminescence signals. The Luc activity was validated in vitro by titrating the cell number, resulting in comparable signal intensity between B6 and CBA Luc-iPSC (Additional file 1: Supplementary Fig. 1). We injected Luc-iPSC into the gastrocnemius muscle of recipient mice, and growing Luc-expressing iPSC-derived teratomas was sequentially monitored for up to 25 days after injection (Fig. 3A). When the iPSCs were injected in syngeneic mice, B6-derived one grew faster to form teratoma than CBA-derived one did (Additional file 1: Supplementary Fig. 5). For controlling immune response in the allogeneic transplantation, TDC + mAb or CB + rapa therapies were conducted similar to Figs. 1B or 2A, respectively. Luminescence signals of iPSC-grafts in non-treatment control groups were significantly lower than that of TDC + mAb or CB + rapa groups on 25 days post-injection, while both immunosuppression regimens allowed Luc-iPSC to gradually grow until the endpoint (Fig. 3B-D). Although we did not observe a significant difference in Luc signal intensity between the two immunosuppressive therapies, teratomas in the TDC + mAb group contained infiltrated immune cells in the vicinity of differentiated tissues, whereas this was not the case in the CB + rapa group (Fig. 3E). We performed MLR using splenocytes from the recipient mice of each treatment group collected later than 25 days post-transplantation. As a result, significant CD4^+^ T cell proliferation was observed against both donors in the TDC + mAb group, as in the skin transplantation (Fig. 3F). We also tested for the presence of donor-specific antibody in the serum of recipient mice treated with TDC + mAb or CB + rapa, but no donor-specific antibodies were detected in both treatment groups (Fig. 3G). Interestingly, we found that frequency of naive (CD44^lo^CD62L^hi^) T cells in the spleen of CB + rapa recipients was relatively higher than that of TDC + mAb ones (Additional file 1: Supplementary Fig. 2). These findings support the robust efficacy of CB-based immunosuppressive therapy in the case of MHC matched/minor antigen mismatched iPSC-based transplantation.

### Co-stimulatory blockade alleviates immune response against human iPSC-derived liver organoids

**Fig. 4:**
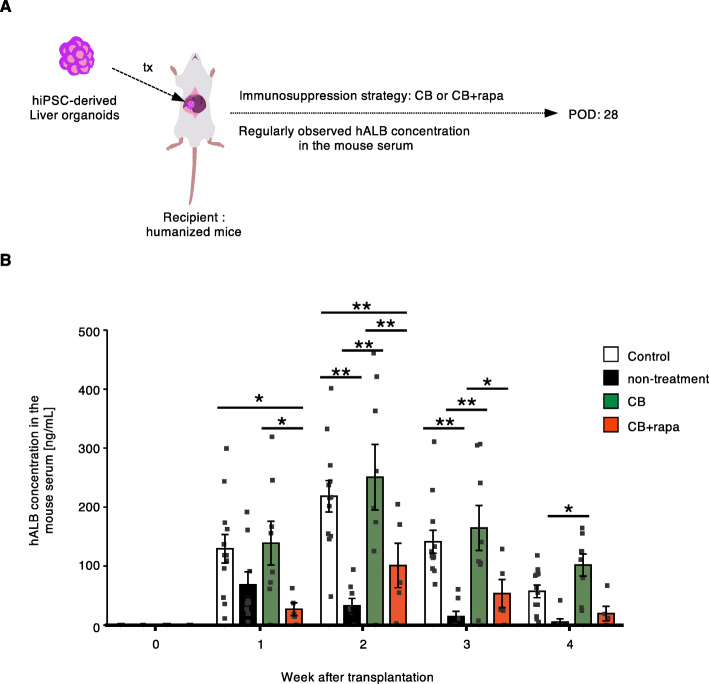
Co-stimulatory blockade alleviates immune response against human iPSC-derived liver organoids. (**A**) Schematic overview of human iPSC-derived liver organoid transplantation. (**B**) Human albumin concentration in the serum of liver organoid recipient mice (*n* = 12, control; *n* = 8-9, non-treatment; *n* = 7, CB; *n* = 5, CB + rapa). *p < 0.05, **p < 0.01 (two-way repeated measure ANOVA Tukey’s multiple comparisons test). hiPSC, human iPSC; tx, transplantation; CB, co-stimulatory molecule blocking; rapa, rapamycin; POD, post-operative day; hALB, human albumin

In order to confirm the efficacy of CB-based treatment on human immune cell reaction to iPSC-grafts, we generated immune humanized mice and transplanted them with human iPSC-derived liver organoids of allogeneic donor origin (Fig. 4A). We have beforehand observed that tacrolimus substantially prolonged the liver organoid survival in this model [38], indicating that T cell-mediated immune reaction is at least partially responsible for the loss of iPSC-derived allogeneic grafts. As tacrolimus could solely regulate the rejection as described above, TDC-based treatment could not be evaluated with this model. Thus, in this study, we focused on the therapeutic potential of CB-based treatment. Humanized mice generation and liver organoid transplantation were carried out following procedures we have previously described [38]. Given that one of the reported limitations of the humanized mice is an insufficient lymphoid immune response due to poor lymph node development [39, 40], we used HLA haplotype A mismatched combination in this experiment. In brief, highly immunodeficient NOG mice were injected with human HLA-A2^+^ hematopoietic stem cells, and the reconstitution of human immune cells was confirmed by FACS analysis of the peripheral blood (Additional file 1: Supplementary Fig. 3). Then, allogeneic human liver organoids derived from HLA-A24^+^ homozygous iPSC were transplanted onto the liver surface of the immune humanized mice. Consistent with our previous report, survival of the liver organoids was gradually decreased in the non-treatment group, which was traced by detecting human albumin concentration in the recipient mouse serum, compared with the control group which was not reconstituted with human hematopoietic stem cells (Fig. 4B). On the other hand, the level of human albumin was continuously comparable between CB-treated and control groups until the study endpoint (Fig. 4B). We found that, unexpectedly, maintenance rapamycin treatment worsened the organoid survival. The adverse effect by rapamycin in this setting may be explained by a liver-damaging effect of mTOR1 inhibition and may discourage using rapamycin for the liver organoid transplantation [41].

## Discussion

Our skin transplantation model revealed diverse susceptibility to immunological rejection among minor antigen-mismatched donors from mild to severe, showing distinct responses to immunosuppression regimens with TDC or TDC + mAb, regardless of their general use in clinical settings. In the case of severe combination, represented by B6 in this study, TDC-based therapy could not efficiently regulate the immune response against the skin and iPSC-derived grafts (Figs. 1 and 3E). These observations may give rise to an important argument in the process of developing an immune regulatory strategy for allogeneic iPSC-based transplantation. Particularly considering that graft rejection in cellular or tissue transplantation, which is generally thought to be performed in iPSC-based transplantation, is relatively difficult to regulate, an alternative approach other than TDC-based treatment would be required. In this study, we have shown that CB treatment, particularly combined with rapamycin, is effective to regulate the immune response to not only mild but also severe risk donor-derived skin (Fig. 2) or iPSC (Fig. 3) grafts with only a few doses of CB in the early stages of transplantation. In the experiment of allogeneic human iPSC-derived liver organoid transplantation (Fig. 4), the efficacy of CB treatment was basically confirmed. Similar indications were provided previously that CB treatment enhanced MHC-mismatched mouse iPSC-graft survival through the downregulation of T cell activation gene signatures [27]. Thus, our present study strongly suggests a limited effect of TDC but a potent inhibitory effect of CB on iPSC-derived allograft rejection, even in MHC-matched but minor antigen-mismatched situations.

A major impediment of clinical translation of CB therapy is that conventional anti-CD40L mAb is currently not available due to prothrombotic toxicities [42]. Recently, a collaborative work by Viela Bio, AstraZeneca, and MedImmune demonstrated that novel CD40L-targeting non-antibody scaffold protein did not induce platelet aggregation in vitro and improved disease activity of patients with autoimmunity without any complications [43]. Such technical advances will support the future application of CB therapy in clinical practice.

It is worth establishing that a method to examine whether the donor iPSC and recipient combination is at risk of severe rejection. If this was possible, mild combination iPSC could be selected, and conventional TDC-based therapy would be a choice to control rejection. Furthermore, the type of cells or tissues derived from iPSC would determine the levels of immune responses, as transplanted grafts from different origin exert various levels of immunological rejection, e.g., rodent skin, small bowel, and lung transplantation are suffered from much severer rejection, whereas heart, kidney, and liver are progressively more easily accepted [44–48]. Therefore, when cells or tissues were generated from iPSC for transplantation, immunological characteristics of them should be carefully estimated before the transplantation. In this regard, evaluating the response of recipient immune cells mixed with cellular grafts may be a tool, which demonstrated to reflect the underlying potential of rejection [49]. However, further methodological development is required to make this reliable because we found that simply co-culturing donor and recipient cells was not sensitive enough to distinguish mild and severe combinations in MHC-matched pairs [17]. Even in this case, attention should be paid to signatures of rejection onset as CD4^+^ T cell proliferation and antibody production were detected in vitro analysis of TDC therapy recipients.

The findings reported in this article provide clear evidence of various rejection severity and response to immunosuppression in MHC-matched and minor antigen mismatched transplantation. To date, the modality of immunosuppression is not limited to traditional chemical compounds or antibody-based strategies, but cellular treatment is becoming an alternative option [50]. Since this study did not evaluate the efficacy of the cellular immunosuppressive intervention, the degree of its effect on severe and mild rejection caused by minor antigen mismatch remains to be elucidated. A better understanding of immunological issues and further development of conventional and state-of-the-art technologies of regulating the immune system are of great importance to expedite the clinical application of iPSC-based therapy.

## Conclusions

Minor histoincompatibility is one of the limitations of successful transplantation, which may hinder the clinical application of MHC-homozygous iPSC. The immunological rejection caused by minor antigen-mismatch ranged from mild to severe. Conventional drug-based immunosuppression showed a limited effect as it could control only mild rejection when combined with T cell depletion. On the other hand, CB therapy successfully prevented rejection regardless of the rejection severity. These results suggest the importance of carefully evaluating rejection and immunosuppressive therapy in MHC-matched iPSC transplantation.

## Supplementary Information

**Additional file 1: Supplementary Fig. 1.** Comparison of CBA/N or C57BL/6 iPSC number with light emission signal. Luciferase expression level in each cell line was measured using SYNERGY4 luminescent detection reader with Gen5 software. Luc, luciferase. **Supplementary Fig. 2.** Naive and memory T cell frequencies in the spleen of iPSC-graft recipients. Flow cytometry analysis (POD 32) of memory (CD44hiCD62Llo/hi) and naive (CD44loCD62Lhi) T cell frequency in the spleen of recipient (C3129F1) mice transplanted with CBA/N or C57BL/6 Luc-iPSC. Luc, luciferase; TDC, three drug combination; mAb, monoclonal antibody; CB, co-stimulatory molecule blocking; rapa, rapamycin. **Supplementary Fig. 3.** Human hematopoietic cell chimerism in the humanized NOG mice peripheral blood. hCD45 chimerism (left-top) (n = 16), hCD33^+^ cells (middle-top) (n = 16), hCD19^+^ cells (right-top) (n = 16), hCD3^+^ cells (left-bottom) (n = 16), hCD4^+^ cells (middle-bottom) (n = 21), and hCD8^+^ cells (right-bottom) (n = 21). hALB, human Albumin. **Supplementary Fig. 4.** Mouse skin graft survival and body weight changes of recipient mice over time under each immunosuppressive regimen. (A-D) were rearranged from the data shown in Fig. 1C, D, and 2 B, and C. (A-D) Skin graft survival under various immunosuppression (n > 8). *p < 0.05 (Two-way repeated-measurement analysis of variance (ANOVA) followed by Holm–Sidak’s multiple comparison test). (E-H) Body weight changes of recipient mice over time under various immunosuppression. n = 9 per group. Normalized to POD-1 weight. **Supplementary Fig. 5.** Bioluminescence images of C57BL/6 or CBA/N Luc-iPSC transplanted into syngeneic mice. The mice were untreated. n = 3 per group.

**Additional file 2: Supplementary Table 1.** Antibody list for flow cytometry

## Data Availability

All data generated or analyzed during this study are included in this published article [and its supplementary information files].

## Abbreviations

iPSC: iPS cells
CB: Co-stimulatory molecule blockade
MHC: Major histocompatibility complex
HLA: Human leukocyte antigen
NOG: Nonobese diabetic/Shi-scid
BLI: Bioluminescent imaging
PI: Propidium iodide
DAPI: 4′,6-diamidino-2-phenylindole
HSD: Honestly significant difference
F1: C3129F1
B6: C57BL/6 J
CBA: CBA/N
Tac: Tacrolimus
MMF: Mycophenolate mofetil
MP: Methylprednisolone
TDC: Triple drugs combination
MST: Median survival time
HE: Hematoxylin and eosin
mAb: Monoclonal antibodies
MLR: Mixed lymphocyte reaction
CFSE: Carboxyfluorescein diacetate succinimidyl ester
CD40L: CD40 ligand
APC: Antigen-presenting cell
rapa: Rapamycin
Luc: Luciferase
