# Supplementary material for: Evaluation of immunosuppression protocols for MHC-matched allogeneic iPS cell-based transplantation using a mouse skin transplantation model
Source: Inflamm Regen. 2022 Feb 2;42:4. doi: 10.1186/s41232-021-00190-7 (PMC8809003; doi:10.1186/s41232-021-00190-7)

Supplemental Figure 1

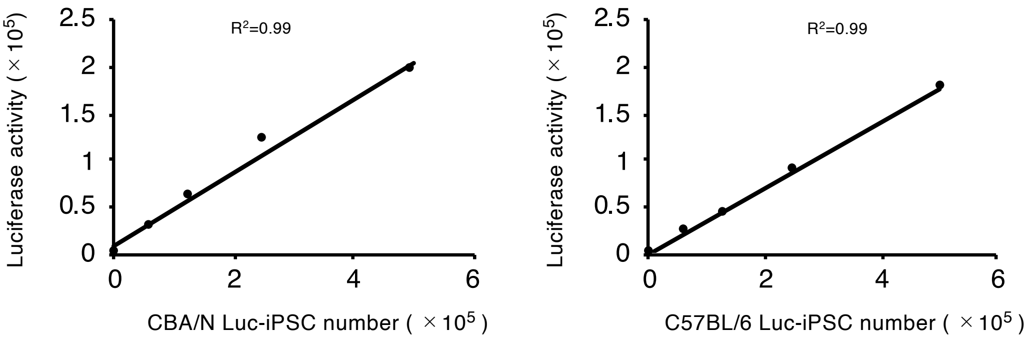

Supplemental Figure 2

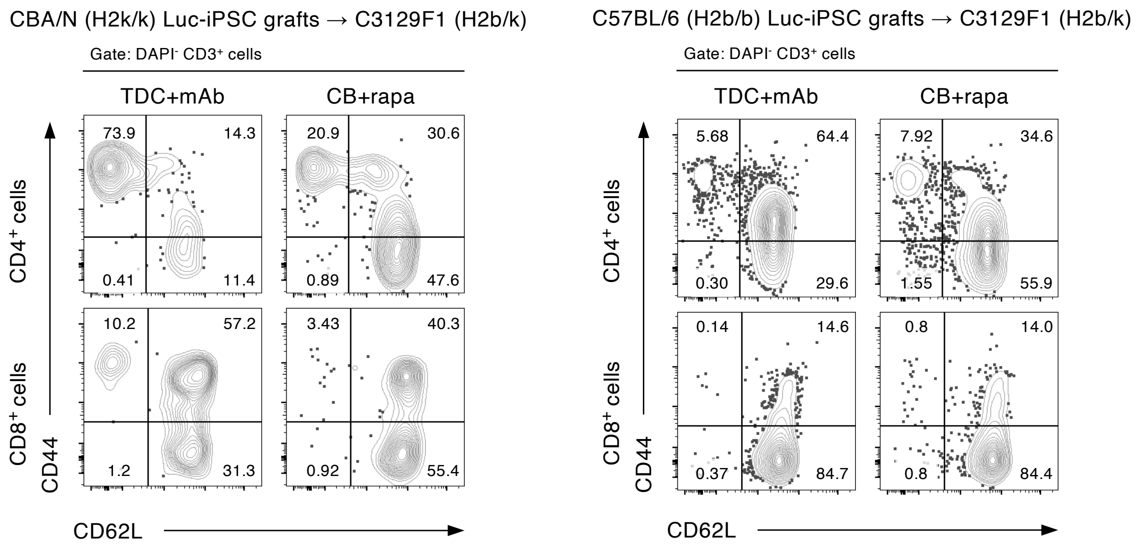

Supplemental Figure 3

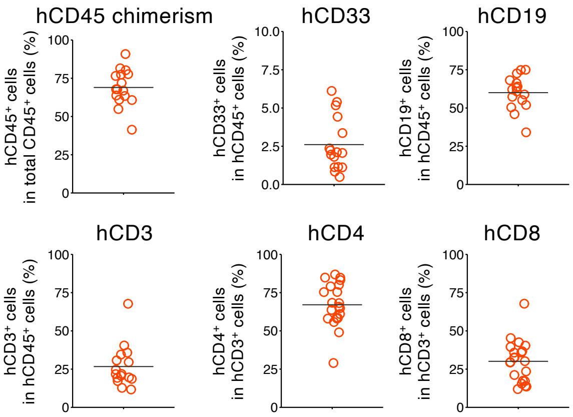

Supplemental Figure 4

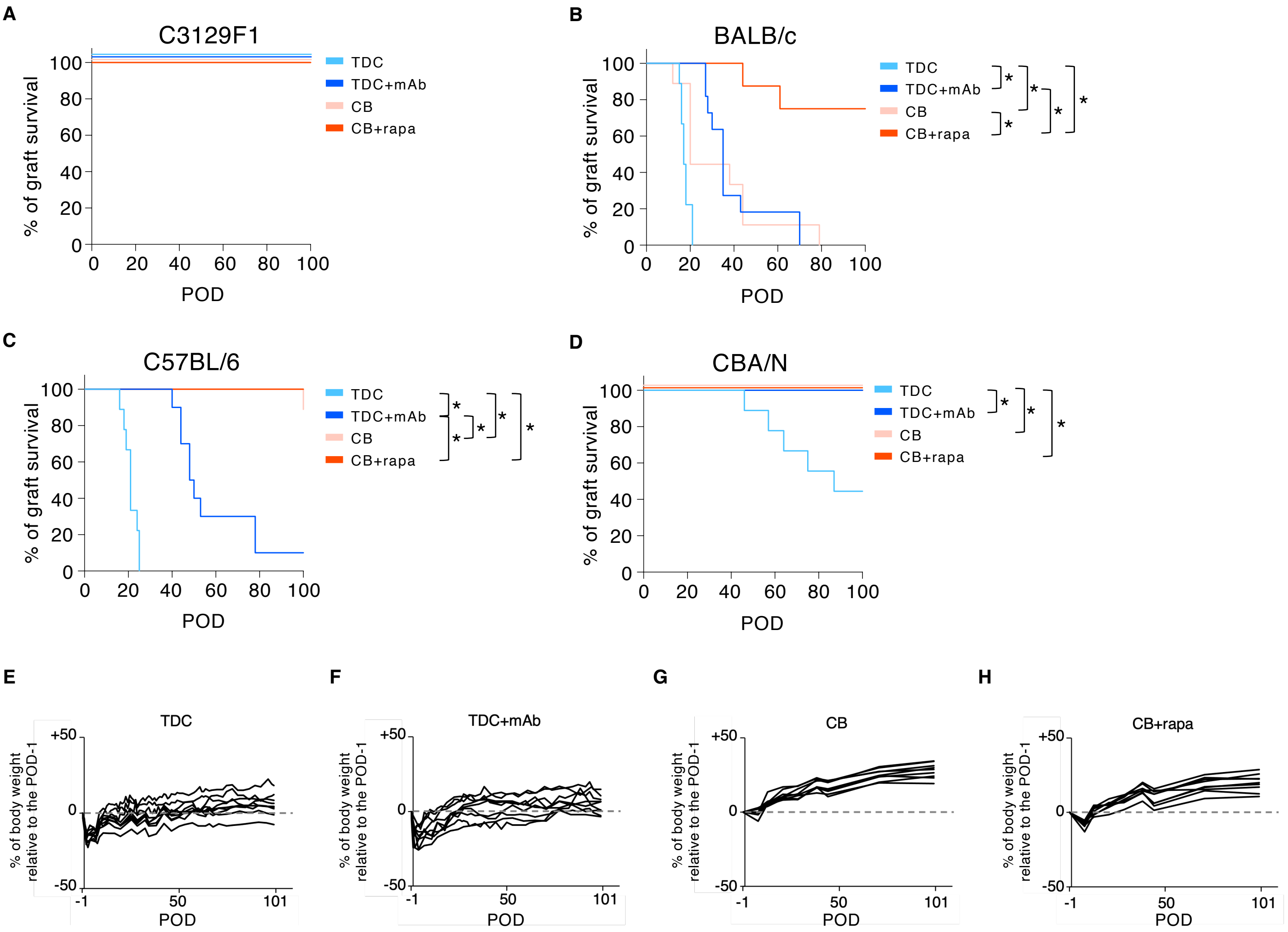

Supplemental Figure 5

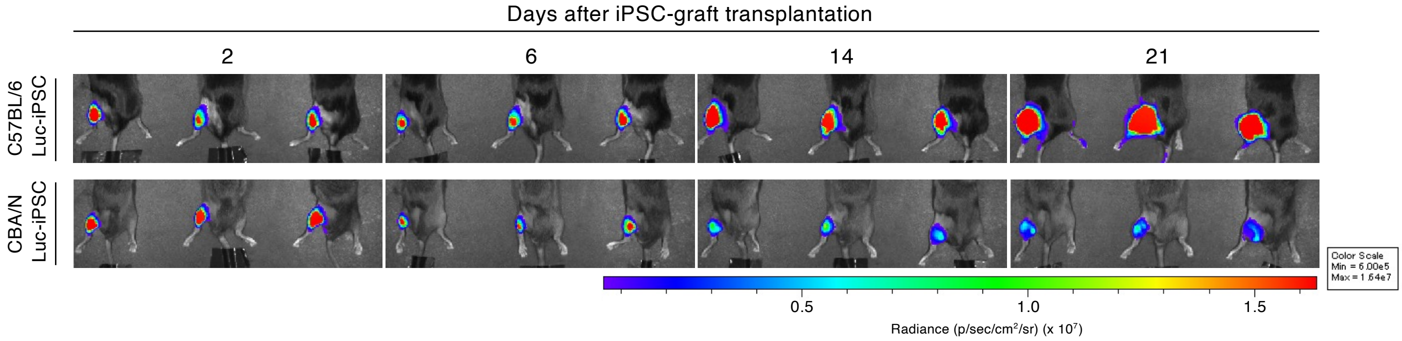

Supplement: Supplementary file 1 — Additional file 1: Supplementary Fig. 1. Comparison of CBA/N or C57BL/6 iPSC number with light emission signal. Luciferase expression level in each cell line was measured using SYNERGY4 luminescent detection reader with Gen5 software. Luc, luciferase. Supplementary Fig. 2. Naive and memory T cell frequencies in the spleen of iPSC-graft recipients. Flow cytometry analysis (POD 32) of memory (CD44hiCD62Llo/hi) and naive (CD44loCD62Lhi) T cell frequency in the spleen of recipient (C3129F1) mice transplanted with CBA/N or C57BL/6 Luc-iPSC. Luc, luciferase; TDC, three drug combination; mAb, monoclonal antibody; CB, co-stimulatory molecule blocking; rapa, rapamycin. Supplementary Fig. 3. Human hematopoietic cell chimerism in the humanized NOG mice peripheral blood. hCD45 chimerism (left-top) (n = 16), hCD33+ cells (middle-top) (n = 16), hCD19+ cells (right-top) (n = 16), hCD3+ cells (left-bottom) (n = 16), hCD4+ cells (middle-bottom) (n = 21), and hCD8+ cells (right-bottom) (n = 21). hALB, human Albumin. Supplementary Fig. 4. Mouse skin graft survival and body weight changes of recipient mice over time under each immunosuppressive regimen. (A-D) were rearranged from the data shown in Fig. 1C, D, and 2 B, and C. (A-D) Skin graft survival under various immunosuppression (n > 8). *p < 0.05 (Two-way repeated-measurement analysis of variance (ANOVA) followed by Holm–Sidak’s multiple comparison test). (E-H) Body weight changes of recipient mice over time under various immunosuppression. n = 9 per group. Normalized to POD-1 weight. Supplementary Fig. 5. Bioluminescence images of C57BL/6 or CBA/N Luc-iPSC transplanted into syngeneic mice. The mice were untreated. n = 3 per group. [file 41232_2021_190_MOESM1_ESM.pdf]
